# Supplementary figures and images for: Basigin mediation of Plasmodium falciparum red blood cell invasion does not require its transmembrane domain or interaction with monocarboxylate transporter 1
Source: PLoS Pathog. 2024 Feb 5;20(2):e1011989. doi: 10.1371/journal.ppat.1011989 (PMC10868855; doi:10.1371/journal.ppat.1011989)

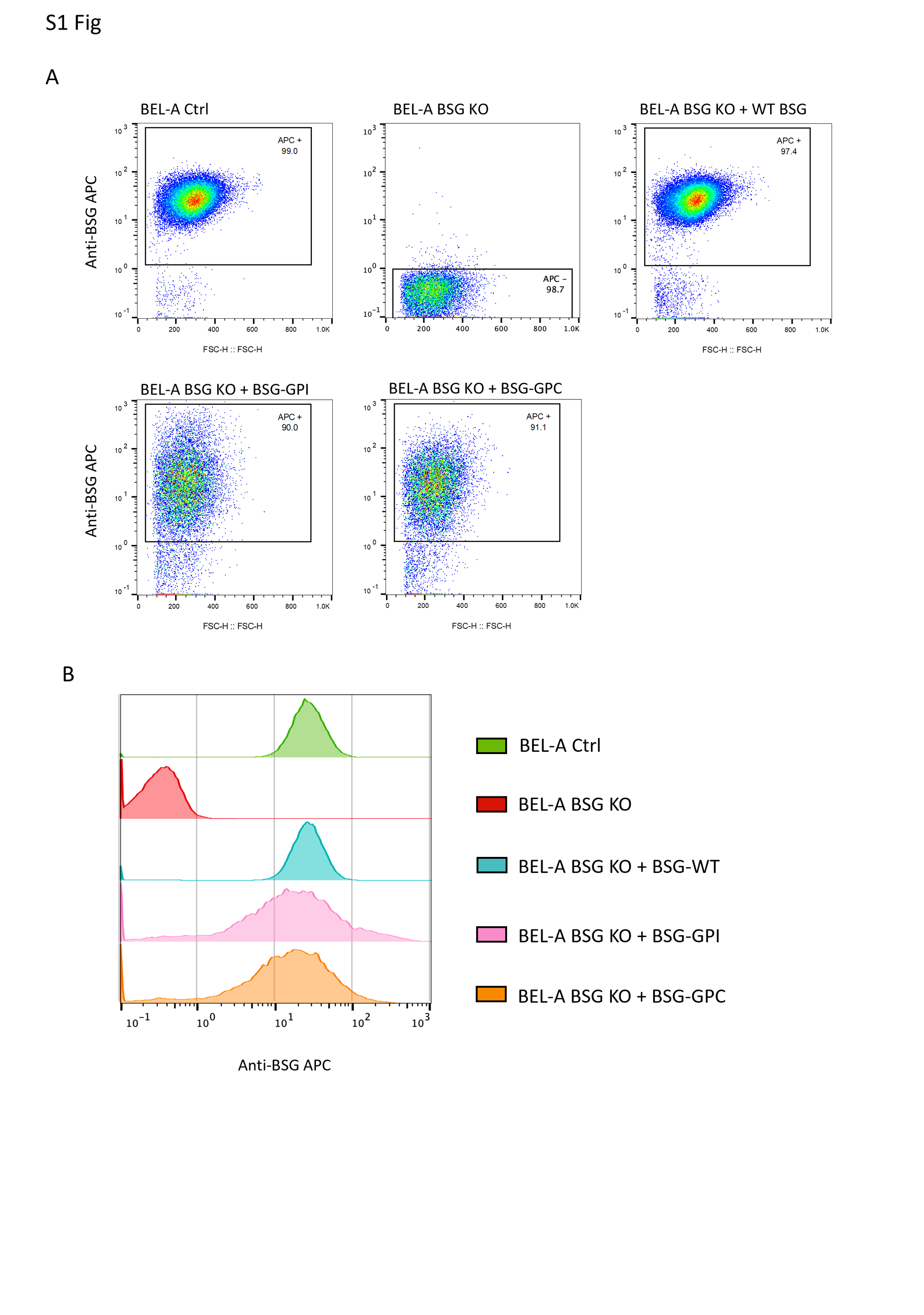

Supplement: S1 Fig — A) Flow cytometry dot plots illustrating surface basigin expression of BEL-A derived reticulocytes as indicated B) Flow cytometry histograms illustrating comparative surface expression of basigin as assessed by HIM6 labelling on BEL-A derived reticulocyte populations. (TIF) [file ppat.1011989.s001.tif]

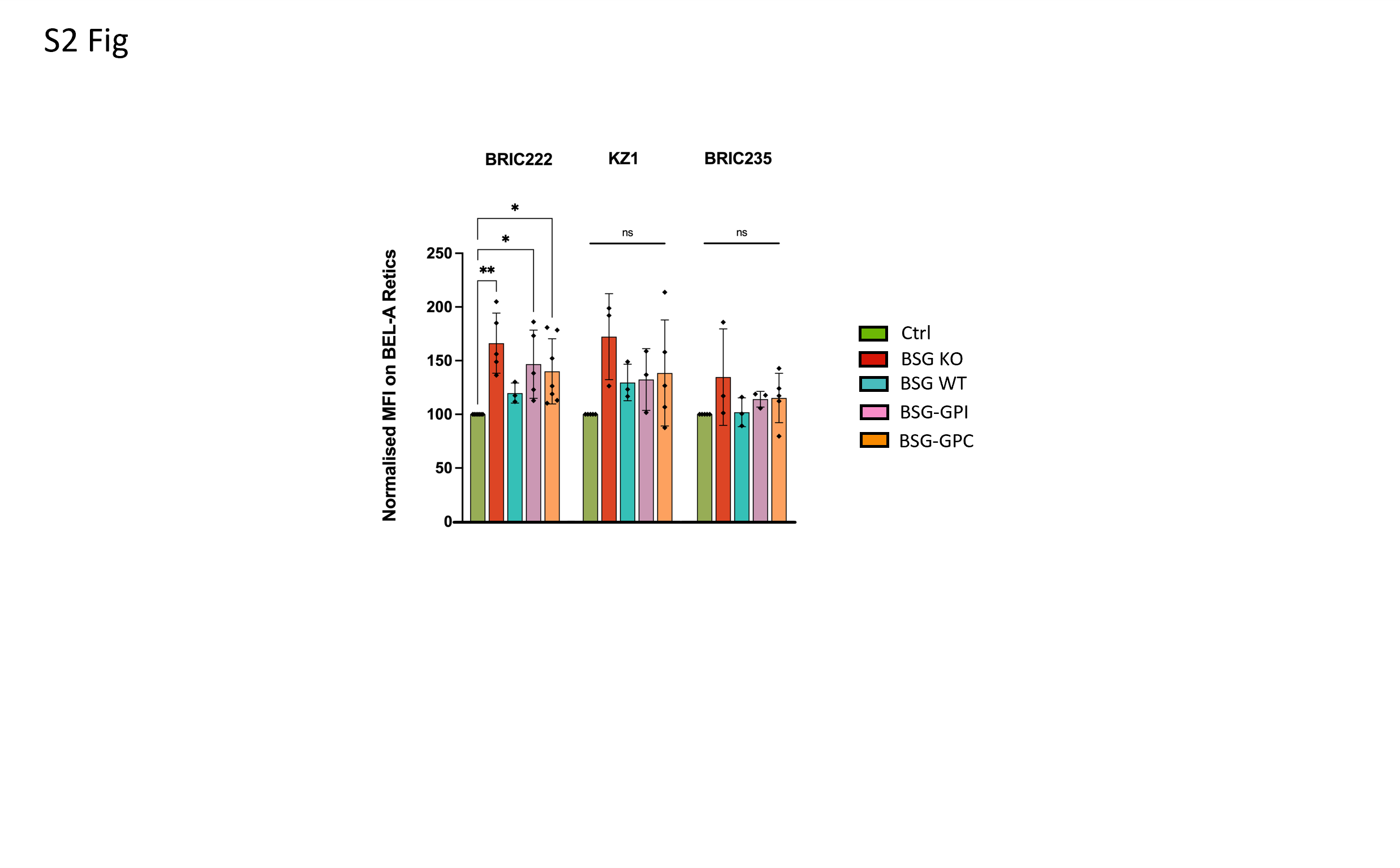

Supplement: S2 Fig — Bar chart illustrating expression of CD44 as assessed with anti-CD44 BRIC222, KZ1 and BRIC235 on indicated BEL-A derived reticulocytes normalized to unedited cells. Data from at least 3 independently differentiated experiments are presented. A Kruskal-Wallis comparison followed by Dunn’s multiple comparison correction was performed to test for differences between groups. p < 0.05 was considered statistically significant. (TIF) [file ppat.1011989.s002.tif]

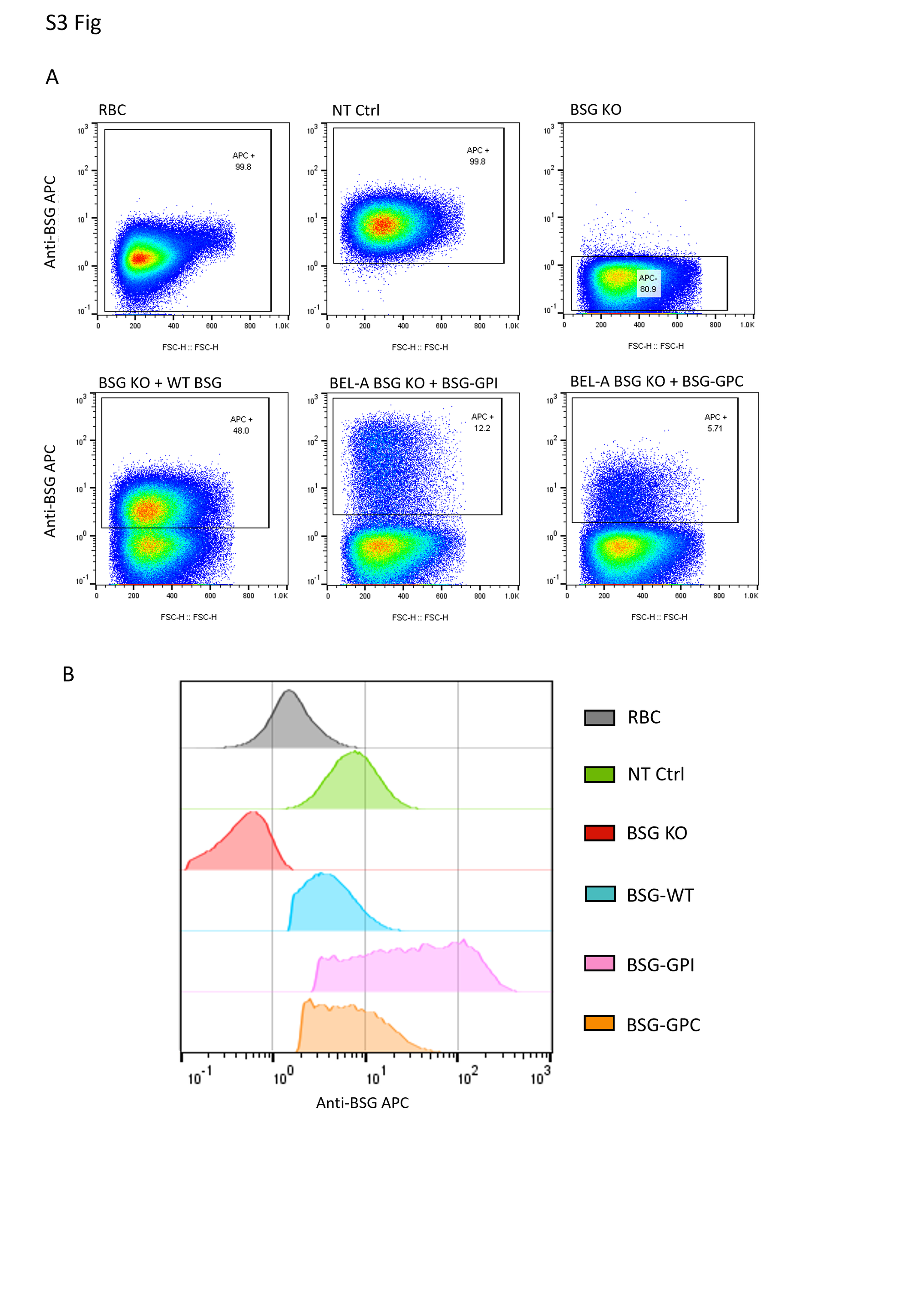

Supplement: S3 Fig — A) Flow cytometry dot plots illustrating basigin surface expression in BSG KO and rescue populations as assessed by HIM6 labelling B) Flow cytometry histograms illustrating basigin surface expression of successfully rescued populations by comparison to RBC, NT Ctrl reticulocytes and BSG KO. (TIF) [file ppat.1011989.s003.tif]
