# Supplementary material for: Basigin mediation of Plasmodium falciparum red blood cell invasion does not require its transmembrane domain or interaction with monocarboxylate transporter 1
Source: PLoS Pathog. 2024 Feb 5;20(2):e1011989. doi: 10.1371/journal.ppat.1011989 (PMC10868855; doi:10.1371/journal.ppat.1011989)
Supplement: Attachment — Submitted filename: King et al Response to Reviewers.pdf [file ppat.1011989.s005.pdf]

Point by Point Response to Reviewers

Part I - Summary
Please use this section to discuss strengths/weaknesses of study, novelty/significance, general execution and scholarship.

Reviewer #1: This paper presents an interesting and compact study in which the authors employ their expertise in genetic manipulation of primary haematopoietic stem cells, which can be invaded by the malaria parasites, Plasmodium falciparum, to assess the importance of the transmembrane domain of the receptor basigin. This is an interesting question as basigin is required for the invasion of erythrocytes by this parasite, and a variety of theories exist about how this happens. These include signalling through basigin, which the authors have already tackled by replacing basigin with a version lacking its cytoplasmic domains, in a previous publication. Basigin also forms membrane complexes with the membrane proteins PMCA and MCT1, through their transmembrane helix. The authors here replace the transmembrane helix of basigin with that of a different protein or with a GPI anchor and assess the effect on invasion. It appears as though this change has blocked the binding of basigin to MCT1 (as would be expected based on the structure) due to the change in MCT1 surface expression which occurs as a result. They then proceed to show that versions of basigin which only have the basigin ectodomain with a different TM or GPI can complement WT basigin, forming reticulocytes which can be invaded by parasites. This is a novel experiment and an interesting outcome as it throws into question all of the models in which basigin acts through transmembrane interactions with other proteins and/or signalling into the erythrocyte. In summary, this is a highly focused paper, with a single key experiment, repeated with two different knockout lines in a robust manner. It gives a clear conclusion, with important consequences for a major malaria vaccine immunogen.

Reviewer #2: Plasmodium falciparum parasites use their RH5 protein to bind to the RBC receptor basigin (BSG) to facilitate invasion of human RBCs. RH5 is attached to the surface of the invasive merozoite parasite stage via a chain of 4 other proteins collectively known as the PCRCR complex. BSG is anchored to the RBC surface via a transmembrane domain (TMD) and binds to either of two other RBC proteins called MCT1 and PMCA1/4. During invasion a calcium ion flux forms at the synapse between the merozoite and RBC leading to the proposal that there may be some rearrangement of the RH5/BSG complex which involves BSG's transmembrane domain and results in a pore in the RBC membrane. BSG's interaction with MCT1 facilitated by BSG's TMD, might also have an important role in invasion. To explore this further, the authors modify erythroblasts to knockout BSG or replace it with chimeric versions of BSG in which its membrane anchor has been replaced with that of another protein or with a GPI anchor. As anticipated, BSG knockout reticulocytes could not be invaded but retics with the alternative membrane anchored forms of BSG can be invaded despite not binding MCT1. Overall, the paper shows that only the ectodomain of BSG is needed for successful parasite invasion of RBCs.

Reviewer #3: In this study the authors are investigating molecular details of basigin-MCT1 interaction in the context of Plasmodium falciparum invasion of human erythrocytes. Erythrocyte invasion is a crucial and mandatory step for the proliferation of the malaria parasite. The multi-step process involves a complex and still only partially understood molecular machinery. It is mediated by receptor-ligand interaction that allows this cell-cell interactions. In P. falciparum it comprises parasite ligands such as the P. falciparum erythrocyte binding-like (PfEBL) and reticulocyte binding protein homologue (PfRh) protein families as well as erythrocyte surface structure such as glycophorin A or basigin. Individual disruption of the PfRh- and PfEBL-encoding genes does not result in an erythrocyte invasion phenotype exposing functional redundancy within these receptor–ligand interactions - with one exception: PfRH5 is the only member of either family showns to be necessary for erythrocyte invasion

in all tested parasite strains. PfRh5 forms a complex with the parasite Ripr (Rh5 interacting protein) and CyRPA (cysteine-rich protective antigen) to interact with the erythrocyte membrane protein basigin. Basigin itself is part of a multiprotein complexes that includes the monocarboxylate transporter MCT1.

Following up on some of their previous work the authors present an elegant study that feeds on genetically manipulated in vitro derived reticulocytes expressing different Basigin variants. The authors demonstrate that merozoite invasion of reticulocytes is unaffected by disruption of basigin MCT1 interaction via various mutations. They conclude, that the MCT1-Basigin interaction is not a requisite for parasite invasion. Overall, the experimental set-up is elegant and well designed and the results are presented clearly. The derived some new insights that challenge some previous assumptions of the complex molecular interplay involved in P. falciparum erythrocytes invasion.

We thank the reviewers for their concise, thorough summaries and positive and enthusiastic reception of our study.

Part II – Major Issues: Key Experiments Required for Acceptance

Please use this section to detail the key new experiments or modifications of existing experiments that should be absolutely required to validate study conclusions. Generally, there should be no more than 3 such required experiments or major modifications for a "Major Revision" recommendation. If more than 3 experiments are necessary to validate the study conclusions, then you are encouraged to recommend "Reject".

Reviewer #1: (No Response)

Reviewer #2: None

Reviewer #3: I have only two major points to discuss:

1. Fig. 1A/B: Quantification of basign and and MCT1 by FACS in mutant cell lines. As a second line of evidence IFA based quantitative microscopy would be nice to probe into the effect of the absence of basigin on reticulocyte surface expression of MCT1 and vice versa. Additional Western Blot or mass spectrometry-based expression analysis could also contribute to a more comprehensive description of the generated mutant cell lines and validate the given FACS-based analysis and the main conclusions. Out of curiosity: Did the authors contemplated on a rescue of the MCT1 KO line on an over expressing BSG parental cell line? Feasible?

We thank the reviewer for their suggestion. Flow cytometry was selected as the most appropriate technique balancing sensitivity, the capacity for single cell expression assessment and the availability of cell material. Since the sensitivity of available antibodies is low, large amounts of cells are required for immunoblotting which given the expense in generating *in vitro* derived mutant cells was not justifiable. IFA based quantitative microscopy provides an alternative to flow cytometry however since the labelling and output is essentially the same as flow cytometry but with lower numbers of cells we do not feel that the additional work required to generate these cells and analyses is necessary to support the conclusions.

Regarding the reviewers suggestion, we found the phrasing of the question slightly confusing but believe that it means did we consider overexpressing WT BSG on an MCT1 KO background to overcome the reduction in endogenous basigin that accompanies MCT1 KO. Since our goal was to more broadly assess the importance of basigin membrane context and lateral interactions for

mediation of invasion we did not perform this experiment but believe that it would be feasible as a means to focus on the effects of loss of MCT1 alone however given the level of effort and expense to generate these cells we feel that the additional information provided would be incremental above that presented here

2. Figure 3+4: Invasion assays.
2.1 What was the rationale for using up to 8% schizonts as starting parasitemia? If egress is normal within their experimental set up it translate to a massive amount of free, invasion capable merozoites although Figure 3A shows a maximum of 8% parasitemia as an end point. Is the invasion capability of 3D7 parasites of the engineered cells that low? Why? What are the implications? Is it also parasite strain dependent?

In our invasion assay setups conducted in 96 well plates, the parasite multiplicity ratio observed is typically 1-2 but sometimes lower depending on the quality of magnet purified schizont prep for a given invasion assay. We have previously observed ratios of this level (which we find to be similar for both donor erythrocytes and in vitro cultured reticulocytes in this setup). We therefore assume and previously suggested that this reflects the low haematocrit brought about by the practical necessity to limit target cells to 0.5-1x10^6 (compared to 5% haematocrit used for routine cultures or invasion studies where cell numbers are not limited). We do not therefore believe there are implications for the results (which include technical and biological replicates including using different parasitemias), or believe that there are strain dependent implications where exploitation of basigin (established to be parasite strain transcendently essential) is being assessed.

2.2 Some additional documentation of the invasion assays by giemsa stained smears at multiple time points (e.g. schizont, post egress, end point) showing for instance normal and complete egress of the used schizont material, the presence of rings (and a substantial amount of non-invaded merozoites) after reinvasion would give the reader another possibility to assess this invasion assay.

Generation of the cells exploited for this study is both challenging and expensive. In order to maximise data acquisition from small numbers of cells whilst also enabling successfully rescued cells to be discriminated from KO by co-labelling with anti-basigin, all cells were used for flow cytometry and there was no material left to do additional experiments. Therefore we unfortunately cannot provide Giemsa stained smears of the sort requested for the experiments conducted in the manuscript.

2.3 As shown in Figure 4B, there are multiple infections with up to five parasites. Is this again due to the “reticulocyte nature” of the host cells? In comparable invasion assays using “normal erythrocytes” multiple infection happen but are rather the exception than the rule.

Indeed the reviewer is correct that the selectivity index (proportion of invaded cells where more than one ring is observed) is high in reticulocytes (this has been reported for both CD34+ and BEL-A derived reticulocytes in our previous study (Satchwell et al, Nature Communications 2019) and by others in alternative systems (Kanjee et al, PNAS 2017).

2.4 Arising from this: Does the used flow cytometry approach i) is able to differentiate between single and multiple infections, ii) are cells with multiple infections quantified as one invasion event? and iii) is the number of multiple infections comparable between the different conditions/cell lines? This appears relevant if the phenotypic consequences of the different variants are assessed.

In answer to the specific points raised i) Flow cytometry is able to distinguish between singly and multiply infected cells (where invasion levels and number of cells acquired is high enough to assign

reliable gates) however this is not a metric that we have focused on (see below) ii) We assessed parasitemia, thus the capacity of a cell to support invasion (whether of one or multiple parasites) has been quantified and cells with multiple rings are counted as one invaded cell iii) Quantifying multiply vs singly invaded cells reliably requires large numbers of invasion events to be assessed across multiple experiments to draw firm conclusions however based on the data available we did not observe differences in the number of multiply infected cells between the different lines.

Part III – Minor Issues: Editorial and Data Presentation Modifications

Please use this section for editorial suggestions as well as relatively minor modifications of existing data that would enhance clarity.

Reviewer #1: First I think that the title and selling of the paper around the interaction with MCT1 doesn't quite fit the data. The main conclusion is broader than being about MCT1, but instead shows that any function of the TM of basigin is not required for invasion. Basigin also binds to PMCAs in erythrocytes, through its TM domains and it is equally interesting that this interaction is not required for invasion. I did consider asking the authors to assess the effect of their mutant basigins on PMCA surface expression, but decided against, as it is likely that the TM replacements will also disrupt PMCA interactions. However, it would be good to broaden the focus of the paper to include discussion of PMCA and signalling too. In fact I would change the title to broaden it from MCT1 to all the other effects of TM changes.

We agree with the reviewer that the conclusions of the paper logically extend beyond the TM domain mediated interaction of basigin with MCT1 alone to encompass other recently or as yet established interactions or functions of this domain and that this is important. Since validation of the disrupted interaction is through the lens of MCT1 and this is the most recognised interaction of this protein we have retained MCT1 within the title but extended it to read "**Basigin mediation of *Plasmodium falciparum* red blood cell invasion does not require its transmembrane domain or interaction with monocarboxylate transporter 1"**

An additional paragraph (below), highlighting the implications of the results for PMCA involvement in invasion has also been added to the discussion.

In addition to complexes with MCT1, basigin has also been reported to exists as heteromeric complexes with PMCA4 (Jamwal et al, 2023), a plasma membrane calcium ATPase required for cellular efflux of calcium in red blood cells, with this interaction also mediated via the transmembrane domain of basigin. Single nucleotide polymorphisms in the ATP2B4 gene that encodes PMCA4 have been associated with resistance to severe malaria in several Genome Wide Association Studies (Ndila et al, 2018, Malaria Genomic Epidemiology Network, 2014, Timmann et al 2012) leading to widespread interest in the putative contribution of this protein in *P. falciparum invasion*, growth and pathogenesis. However, while the identification of a calcium ATPase as interacting partner of basigin (engagement of which by PfRh5 is accompanied by a transient pulse of calcium during merozoite invasion (Volz et al, 2016) provided initial excitement, subsequent data do not support functional modulation of PMCA activity based upon PfRh5 interaction with basigin (Jamwal et al, 2023). Whilst we did not explore effects of basigin TM domain removal on PMCA directly, absence of the effect on invasion of deletion of this interface, as in the case of MCT1, by extension excludes a requirement for direct interaction between basigin TM domain and PMCA proteins for successful *P. falciparum* invasion.

The major omission was the FACS data showing that the different basigin variants are expressed properly and surface exposed. Figure 2B could do with better labelling and would be valuable to see equivalent data in SI for each of the mutants. I presume that there is no issue here as the basigin

variants were invaded by parasites, but it is important to present these validations of correct surface exposure of the variant basigins for both cell types.

Expression of the different basigin mutants in BEL-A derived reticulocytes is presented in the flow cytometry histogram in Figure 1D and shows populations with similar levels of surface expression of basigin in reticulocytes expressing the various mutants to unedited cells. However we have added additional supplemental figures showing flow cytometry dot plots and offset histograms for clarity for BEL-A derived reticulocytes (new S1) and reticulocytes derived from transduced primary CD34+ cells (S3). As noted in the text, in all cases surface expression of reticulocytes presenting the basigin ectodomain (whichever version) exceeds that of erythrocytes as would be expected for the larger reticulocytes so basigin ectodomain presentation is in no cases limiting for invasion. Interestingly BSG KO CD34+ cells rescued with the BSG-GPI construct result in a population of reticulocytes with a broad range of basigin ectodomain presentation including some with much higher than that of unmodified reticulocytes, however there is no apparent increase in the invasion efficiency associated with excess basigin presentation indicative that there is no increased susceptibility to invasion resulting from basigin exposure above a threshold reached in reticulocytes.

Reviewer #2: 1. Overall this is a very well-prepared manuscript with no major faults. I would like to know however, if the retics invaded with the chimeric versions of BSG complete their cell cycles and continue to proliferate. This could be done by seeding the newly invaded retics into a dish of normocytes to see if they proliferate.

We thank the reviewer for their kind comment. The reviewer raises an interesting point. We did not perform these experiments due to limited cell numbers used due to keep costs manageable and also because our focus for this manuscript was specifically on invasion. We would not predict that the chimeric basigin constructs themselves would impair growth and egress themselves, however the effect of the massively reduced expression of MCT1 in these cells on parasite growth may be interesting to assess. The additional work required to generate cells to perform these experiments however is beyond the scope for this revision.

2. For reference 1, use the latest WHO World Malaria Report.

Amended as requested. Reference 27 – Farrell et al, has also been updated from the BioRxiV preprint to reflect the recent publication of this work in Nature.

3. Line 61. Should 'bases' be 'basis'?

This line "the bases for the selection of basigin as a key actor for such an essential process are unclear" uses the plural bases to reflect the fact that there may be multiple.

4. Please clarify what is meant on line 350 '…may be important for invasion within a cellular context.'

This line reflects the fact that the Jamwal et al data discussed illustrates the relevance of BSG as existing as part of a complex with MCT1 for its interaction with PfRh5 in in vitro studies, the implication being that this association may therefore be relevant within the true 'cellular context' of a red blood cell membrane

Reviewer #3: 1. Most of the figures need some adjustments and harmonization. Font and font sizes are varying within and between figures.

Amended as requested

2. Figure 1D/E – What does the black curve represent?

We apologise for the omission of this from the key. Black line represents IgG1 isotype control labelled cells

3. Figure 3B: y-axis has no legend.

Axis label – % invasion normalised to NT added

4. Figure 4 A/B: What was the starting parasitemia for the shown invasion assay?

8% - this has been added to the legend

5. The authors are referring to the WHO Malaria report 2021. They could update the numbers with a newer report.

Amended as requested

6. Again out of the reviewer curiosity (but it might be also interesting for the general reader): Why did the authors focus their study on MCT1 and not the ATPase PMCA1/4 within the Basigin protein complex?

We thank the reviewer for their interest. Focus on the MCT1 interaction is based largely on the fact that the association between basigin and MCT1 has been established for much longer and much of the work contained within this manuscript began prior to the publication on BioRxiV of the studies of Jamwal et al reporting association between basigin and PMCA1/4. It was further facilitated by the development of the viral RBD reagent for detection of surface MCT1. Since the motivation for the study was basigin focused with MCT1 labelling predominantly validatory of removal of the transmembrane domain we believe that the conclusions regarding the dispensability of the transmembrane and cytoplasmic domain of basigin can still reasonable be extended to cover the interaction between basigin and PMCA in addition to that with MCT1 as suggested by Reviewer 1.
